# Acetylome Analysis in *Vibrio vulnificus* MO6-24/O Reveals Extensive Lysine Acetylation in Carbon Metabolism and Protein Synthesis Pathways: A Pilot Study

**DOI:** 10.3390/pathogens15070718

**Published:** 2026-07-07

**Authors:** Yurong Song, Ming Cheng, Xiaoling Li, Ying Wang, Yulong Zong, Jing Li, Guozhong Chen, Shiyong Chen, Yuan Cao

**Affiliations:** 1 Department of Basic Medical Sciences, The 960th Hospital of PLA, Jinan 250031, China; fcheng345@163.com (Y.S.); 13153199161@163.com (G.C.); 2 Departments of Neurology, The 960th Hospital of PLA, Jinan 250031, China; chengming0624@163.com; 3 School of Marine Science and Engineering, Qingdao Agricultural University, Qingdao 266109, China; lxl01202021@163.com; 4 Department of Pulmonary and Critical Care Medicine, The 960th Hospital of PLA, Jinan 250031, China; babyjinan@163.com; 5 Department of Laboratory Medicine, Taian City Central Hospital, Taian 271000, China; zongyulong88@126.com; 6 State Key Laboratory of Pathogen and Biosecurity, Academy of Military Medical Science, Beijing 100071, China; lj-pbs@163.com

**Keywords:** lysine acetylation, acetylome, lysine acetylation motif, interaction network, carbon metabolism, *Vibrio vulnificus*

## Abstract

*Vibrio vulnificus* (*V. vulnificus*) is a type of bacterium commonly found in estuarine environments. It can cause necrotizing wound infections and sepsis, both of which are associated with high mortality rates. Protein lysine acetylation is a widespread post-translational modification (PTM) of proteins, and it participates in numerous cellular processes, including regulation, in bacteria. However, the finer landscape of lysine acetylation in *V. vulnificus* remains unexplored. In this study, acetylated proteins with low cellular abundance were enriched from *V. vulnificus* MO6-24/O using anti-acetyl-lysine immunoprecipitation and identified using LC-MS/MS, and the acetylation was further confirmed by Western blot analysis. We mapped 2035 lysine acetylation sites to 841 proteins, accounting for approximately 18.5% of the entire protein sequence of *V. vulnificus* MO6-24/O. Comprehensive bioinformatic characterization of the acetylome indicated that lysine acetylation is associated with metabolic regulation, particularly targeting enzymes regulating carbon metabolic functions and biosynthesis. In addition, sequence motif analysis identified two conserved patterns surrounding acetylated lysines: enrichment of lysine or arginine residues at the +4/+5 positions, and a preference for tyrosine, histidine, or phenylalanine residues at the −1/+1 positions. Furthermore, analysis of the protein–protein interaction network indicated that lysine acetylation influences numerous molecular interactions among proteins. Collectively, this acetylome investigation establishes a foundation for future studies aimed at elucidating physiological functions of protein lysine acetylation in *V. vulnificus*.

## 1. Introduction

Post-translational modifications (PTMs) are important in modulating protein function by influencing enzymatic activity, structural conformation, subcellular distribution, and molecular stability. Among these modifications, lysine acetylation is a key reversible process regulated by acetyltransferases and deacetylases. It was initially discovered in histone proteins [1] and is characterized by its dynamic nature [2]. Initially studied in histones, lysine acetylation is now known to modify numerous non-histone proteins and to participate in diverse processes including metabolism, cell cycle regulation, aging, and stress responses [3,4,5,6]. With the rapid advancement of proteomics technology, the ubiquity of lysine acetylation in bacterial systems has been established, and it has been recognized as an important PTM [7,8]. Early bacterial acetylomes in *Escherichia coli* identified around 100–140 modified proteins [9]. Since then, large-scale acetylomes have been reported across many prokaryotes, demonstrating roles for acetylation in metabolism, translation, and protein folding, and highlighting its regulatory impact on metabolic pathways [10,11].

*Vibrio vulnificus* (*V. vulnificus*) is a zoonotic pathogen of great public health concern and aquaculture that can cause primary septicemia through the ingestion of shellfish. The highest mortality rates thanks to this pathogen occur via wound exposure to contaminated seawater [12]. As a Gram-negative bacterium, it ubiquitously inhabits warm (>15 °C) and low-salinity brackish waters worldwide, and proliferates rapidly in warm seasons [13]. The bacterium is classified into three biotypes, with biotype 1 accounting for nearly all documented human infections [14]. *V. vulnificus* virulence is mediated by factors enabling adhesion, immune evasion, and toxin delivery. The MARTX toxin encoded by rtxA induces host cell death through autoprocessing cytotoxic domains. VvhA hemolysin is a pore-forming cytolysin that synergizes with MARTX to exacerbate disease [14].

Additional virulence determinants include OmpU, capsular polysaccharide, flagella, HlyU and type IV pili [15,16,17]. A recent retrospective clinical study of *V. vulnificus* infections conducted in Hainan Province, China (2018–2023) revealed that 90% of local cases occurred from June to September, which coincides with the period when seawater temperatures peak at 28–32 °C [18]. During heatwaves in July and August 2023, eleven severe *V. vulnificus* infections were reported in three eastern U.S. states; four patients developed septic shock and five died [Notes from the Field: Severe *Vibrio vulnificus* Infections During Heat Waves—Three Eastern U.S. States, July–August 2023. MMWR Morb Mortal Wkly Rep]. In South Korea, a total of 1285 confirmed vibriosis cases were documented between 2003 and 2023. An analysis of 761 cases collected from 2003 to 2016 further indicated that the overall case fatality rate reached 33.4% [19,20]. Climate warming has driven the northward expansion of *V. vulnificus* into temperate marine habitats where this pathogen was rarely detected before [21,22,23,24]. *V. vulnificus* senses a spectrum of host-specific environmental cues absent in our in vitro culture [25], including host serum iron driving sepsis via Fur and acetylation pathways [26], unique host osmotic and pH conditions governing colonization and toxin production [27], acetyl–CoA and other host metabolites linking metabolism to acetylation-mediated virulence [28], host immune mediators facilitating immune evasion [29], and microbiota-derived QS signals AI-2/DSF modulating LuxS/LuxO-dependent biofilm and virulence [30].

The *V. vulnificus* strain MO6-24/O is a widely used reference strain with a fully sequenced genome and well-documented virulence characteristics, making it a representative model for investigating the pathogenic mechanisms of *V. vulnificus* [25,31]. It was originally isolated from a patient with septicemia in 1989 [32]. During human infection, *V. vulnificus* perceives multiple host-associated environmental cues including 37 °C body temperature, tissue iron limitation/overload, fluctuating osmolarity and pH, phagocyte-derived ROS, bile acids, mucins and host carbon nutrients; these signals jointly reprogram its central metabolism and modulate the expression and acetylation of virulence proteins to facilitate colonization, immune escape and systemic invasion [33,34,35,36]. Although numerous virulence factors have been mentioned above, their acetylation levels and the global regulatory mechanisms underlying *V. vulnificus* metabolism and pathogenicity remain incompletely understood. As a pervasive post-translational modification, lysine acetylation plays a crucial role in coordinating metabolic pathways and virulence-related processes, making acetylome profiling essential for elucidating these regulatory mechanisms.

Here, we systematically profiled lysine acetylation in *V. vulnificus* MO6-24/O and identified 2035 sites on 841 proteins. Western blotting confirmed acetylation of four critical proteins: the key metabolic enzymes acetyl–CoA synthetase (Acs) and NAD^+^-dependent protein deacetylase CobB (CobB), along with outer membrane protein U (OmpU) and the virulence-associated immunogenic lipoprotein A (IlpA). Functional categorization revealed that many acetylated proteins participate in central metabolism and protein synthesis. Several conserved sequence motifs were identified around acetylated lysines, and interaction-network analysis indicated extensive regulatory potential. Collectively, this acetylome investigation establishes a foundation for future studies aimed at elucidating physiological functions of protein lysine acetylation in *V. vulnificus*.

## 2. Materials and Methods

### 2.1. Bacterial Strains and Culture Conditions

The MO6-24/O strain was initially cultivated on marine agar 2216E (BD Difco, Sparks, MD, USA, catalog number—279110) composed of peptone (5 g/L), ferric phosphate (0.1 g/L), yeast extract (1 g/L), and sea salt (30 g/L) at an incubation temperature of 37 °C. For experimental analyses, an overnight culture was transferred into 1000 mL of marine salt medium at a 1:100 inoculation ratio and allowed to grow with aerobic conditions until the stationary phase was reached. Samples were first collected after 2 h of incubation, corresponding to the early exponential phase (OD_600_ = 0.16), followed by additional collections at 4 h and 8 h, when cultures entered the stationary phase (OD_600_ = 1.8). The samples collected from different growth phases (2 h, 4 h, and 8 h) were pooled into a single preparation for LC-MS/MS analysis.

### 2.2. Protein Extraction and Digestion

Three bacterial samples were combined and pulverized under liquid nitrogen, followed by protein extraction using lysis buffer. Cell disruption was enhanced by three rounds of sonication performed on ice. The lysates were clarified by centrifugation at 4 °C for 10 min, followed by protein precipitation using 15% (*w*/*v*) trichloroacetic acid (Sigma-Aldrich, St. Louis, MO, USA, catalog number—T6399). The obtained protein was first rinsed with pre-cooled acetone (Sigma-Aldrich, St. Louis, MO, USA, catalog number—179124), and then dissolved in a urea solution containing 100 mM of TEAB (pH 8.0, Sigma-Aldrich, St. Louis, MO, USA, catalog number—T7408). The protein solution was reduced with 10 mM DTT (Sigma-Aldrich, St. Louis, MO, USA, catalog number—D0632), alkylated with 20 mM IAA (Sigma-Aldrich, St. Louis, MO, USA, catalog number—I1149), and digested with trypsin (Thermo Scientific/Pierce, Rockford, IL, USA, catalog number—90057) at ratios of 1:50 overnight and 1:100 for an additional 4 h.

### 2.3. Enrichment of Lysine Acetylated Peptides

To enrich for lysine-acetylated (Kac) peptides, the peptides digested by trypsin were dissolved in NETN buffer and then incubated overnight at 4 °C with gentle agitation using antibody-conjugated beads (PTM Biolabs, Hangzhou, China, catalog number—PTM-104). Following incubation, these beads were subjected to washing four times using NETN buffer and then rinsed two times using deionized water. The peptides attached to the beads were then released by employing a solution that contains 0.1% trifluoroacetic acid. The resulting mixture of peptides was subsequently desalted and purified with C18 ZipTips (Millipore/Merck, Burlington, MA, USA, catalog number—ZTC18S096) according to the manufacturer’s instructions, before undergoing analysis via LC-MS/MS.

### 2.4. LC-MS/MS Analysis

The peptides were reconstituted in solvent A (0.1% formic acid in 2% acetonitrile), trapped on a PepMap pre-column, and subsequently resolved on a PepMap RSLC analytical column using an EASY-nLC 1000 system. Separation was achieved with a linear gradient of solvent B from 6% to 35% over 32 min, followed by an increase from 35% to 80% over 8 min, at a flow rate of 280 nL/min. Peptide analysis was performed using a Q Exactive hybrid quadrupole–Orbitrap mass spectrometer coupled to NSI source. The Q Exactive Plus system was run in data-dependent acquisition mode to carry out tandem MS/MS analysis. Full MS scans were acquired with a resolving power of 70,000, and the ten most intense precursor ions with intensities above 2 × 10^4^ were isolated and fragmented using a normalized collision energy of 28%. MS/MS spectra were recorded with an instrumental resolution of 17,500 and a dynamic exclusion window set to 10 s.

### 2.5. Database Search

MS/MS data analysis was analyzed with the MaxQuant software (version 1.4.1.2) [37]. Spectral searches were performed against the *V. vulnificus* MO6-24/O protein database. Trypsin/P was selected for protein digestion, and the maximum number of allowed missed cleavage sites was set to three. Peptide identification parameters included a maximum of four variable modifications and a charge state limit of five per peptide. FDR threshold was maintained below 1% for protein, peptides, and modification sites. Only peptides containing at least seven amino acids were considered for identification. Modification sites with a localization probability of at least 0.75 were regarded as confidently assigned. All remaining MaxQuant parameters were maintained at their default settings.

### 2.6. Validation of Lysine-Acetylated Proteins

Four candidate proteins were cloned and overexpressed in *V. vulnificus* to verify lysine acetylation detected by proteomic analysis. The genes (*acs*, *cobB*, *ompU* and *ilpA*) were amplified from genomic DNA and inserted into the pSCT32 vector using a ClonExpress recombination cloning kit (Vazyme Biotech, Nanjing, China, catalog number—C112). The recombinant plasmids were sequentially introduced into *E. coli* DH5α (TransGen Biotech, Beijing, China, catalog number—CD201), mobilized through *E. coli* SM10 λpir, and conjugatively delivered into *V. vulnificus*. Exconjugants were selected on chloramphenicol (Sigma-Aldrich, St. Louis, MO, USA, catalog number—C0378) and polymyxin E. Relevant plasmids, primers, and strains were listed in Supplementary Tables S1 and S2. Protein expression was induced using IPTG (Sigma-Aldrich, St. Louis, MO, USA, catalog number—I6758); all constructs carried a C-terminal His tag for immunoblotting and purification. His-tagged proteins were enriched with Ni-NTA magnetic beads (Thermo Scientific/Pierce, Rockford, IL, USA, catalog number—88831) and examined by Western blot with pan anti-acetyl antibody (Cell Signaling Technology, Danvers, MA, USA, catalog number—9441S), with HisProbe-HRP confirming expression. Wild-type *V. vulnificus* without plasmids served as the negative control.

### 2.7. Bioinformatics Analysis

Genomic data and annotation files of *V. vulnificus* strain MO6-24/O were retrieved from GenBank (accession No. GCA_000186585.1). The EggNOG database was further employed for functional annotation [38]. The combined annotation datasets were used to screen and analyze lysine protein acetyltransferases and deacetylases.

Gene Ontology (GO) annotation was carried out with UniProt-GOA by translating the identified protein IDs into UniProt IDs and assigning the corresponding GO terms. Proteins lacking UniProt-GOA annotations were analyzed with InterProScan to assign GO functions based on sequence similarity [39]. KEGG annotation was conducted using the KAAS tool, and results were mapped to pathways with KEGG Mapper. GO and KEGG enrichment were assessed by two-tailed Fisher’s exact tests, with multiple testing correction applied using the Benjamini–Hochberg method. Terms with an adjusted *p* value < 0.05 were considered significantly enriched. Sequence motifs around acetylation sites were examined by Motif-X (version 1.2) using ±10 amino acids around each site, with the full protein database as background and default settings. Protein–protein interactions were assessed using STRING [40] with a confidence score ≥0.7, and network visualization was performed using Cytoscape [41].

## 3. Results

### 3.1. Identification of 2035 Lysine Acetylation Sites in V. vulnificus

To elucidate the acetylation-associated enzymes of *V. vulnificus* from a genomic perspective, functional annotation was conducted against the GenBank and eggNOG databases [38]. In total, 50 acetyltransferases and 9 deacetylases were annotated in *V. vulnificus* MO6-24/O (Supplementary Table S3). Subsequent genomic screening uncovered two GNAT superfamily acetyltransferase genes (VVMO6_00674, VVMO6_02877) and one gene coding for the Sir2-family NAD^+^-dependent deacetylase CobB.

**Figure 1:**
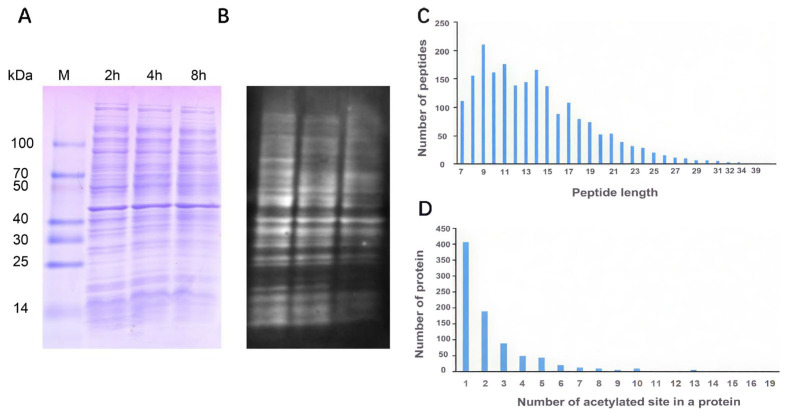
Characterization of lysine acetylation in *V. vulnificus*. (**A**) SDS-PAGE of *V. vulnificus* whole-cell lysate. SDS-PAGE separation of whole-cell protein lysates extracted from *V. vulnificus* samples collected across three growth time points. (**B**) Detection of the overall acetylation of *V. vulnificus*. Western blot detection of total lysine acetylation signals in the whole-cell proteome, verifying abundant acetylation modification at the global protein level of *V. vulnificus*. (**C**) Distribution of acetylated peptides based on their length. Length distribution statistics of all enriched acetylated peptides identified via LC-MS/MS analysis. (**D**) Distribution of acetylated proteins based on their number of acetylation peptides. Quantitative distribution profile of acetylated proteins classified by the number of matched acetylated peptides per protein, reflecting the modification abundance of individual acetylated proteins.

Furthermore, to assess both the range and relative levels of acetylated proteins in *V. vulnificus*, protein extracts were examined by Western blot with pan anti-acetyl antibody. The findings indicated that the acetylation was widespread in *V. vulnificus* at different culture times (Figure 1A,B). Park et al. subsequently completed the whole-genome sequencing of *V. vulnificus* MO6-24/O, providing an essential genomic framework that enables systematic characterization of the lysine acetylome and precise mapping of its modification sites in this organism [31]. We first isolated proteins from pooled cells during growth. Then, an immunoaffinity enrichment-based proteomic approach coupled with high-resolution LC-MS/MS was employed. The mass error distribution was tightly centered around zero, with most values falling below 5 ppm, demonstrating acceptable mass accuracy (Supplementary Figure S1). Most identified peptides were 7–39 amino acids long (Figure 1C), which is in line with the expected characteristics of trypsin digestion.

The acquired MS/MS spectra were matched against the *V. vulnificus* UniProt proteome database (4549 entries) using MaxQuant software. In total, 2035 acetylated peptides with scores exceeding 30 were identified, corresponding to 841 acetylated proteins, each harboring between one and nineteen distinct lysine acetylation sites (Figure 1D, Supplementary Table S4). The pyruvate dehydrogenase E1 component (VVMO6_00532) exhibited the largest number of lysine acetylation sites (n = 19) among all identified acetylated proteins in *V. vulnificus*. Collectively, these findings indicate that lysine acetylation is widespread and potentially plays roles in multiple cellular functions during bacterial growth. Acetylated proteins constitute 18.5% of the *V. vulnificus* MO6-24/O proteome (Figure 1D). While this proportion is lower than in strain Vv180806, it remains higher than levels observed in other bacteria, such as *E. coli* and *V. parahaemolyticus* [42,43]. The relative abundance of lysine acetylation underscores its potential significance in the physiological regulation of *V. vulnificus*.

### 3.2. Validation of Lysine-Acetylated Proteins Using Affinity Chromatography and Western Blotting

**Figure 2:**
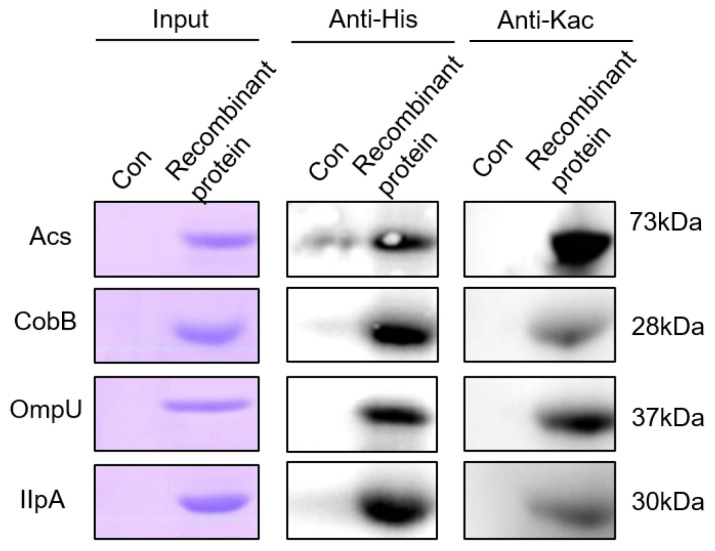
Validation of selected lysine-acetylated proteins in *V. vulnificus*. Polyhistidine-tagged recombinant proteins were enriched from *V. vulnificus* whole-cell lysate with magnetic beads. Candidate proteins were first analyzed by SDS-PAGE, followed by sequential Western blotting detection with Hisprobe (middle panel) and anti-acetyl lysine antibodies (right panel). Wild type *V. vulnificus* strain without recombinant plasmids was used as the negative controls to exclude non-specific binding signals in experiments. Coomassie staining was used as lane inputs (left panel) for each sample. Kac represents lysine acetylation.

For subsequent validation and functional investigation, we preferentially selected proteins with established roles in metabolism and virulence or host interaction to facilitate biological interpretation. Specifically, Acs and CobB are key components of central metabolic pathways and acetylation-mediated regulatory networks, whereas OmpU and IlpA are outer membrane proteins implicated in virulence and host interaction [21,22,23]. These four key genes were therefore selected, cloned, and expressed in *V. vulnificus* with a polyhistidine epitope tag for further experimental validation. The target proteins were enriched through affinity purification using magnetic beads, and the samples were subsequently examined using Western blot analysis with antibodies specific for acetylated lysine residues. A HisProbe-HRP conjugate against the polyhistidine tag was used as a positive control. The results demonstrated that lysine acetylation signals were detectable in the purified proteins with anti-acetyl-lysine antibodies, supporting the accuracy and reliability of the acetylome dataset (Figure 2).

### 3.3. Functional Analysis and Subcellular Distribution of the Lysine Acetylome of V. vulnificus

**Figure 3:**
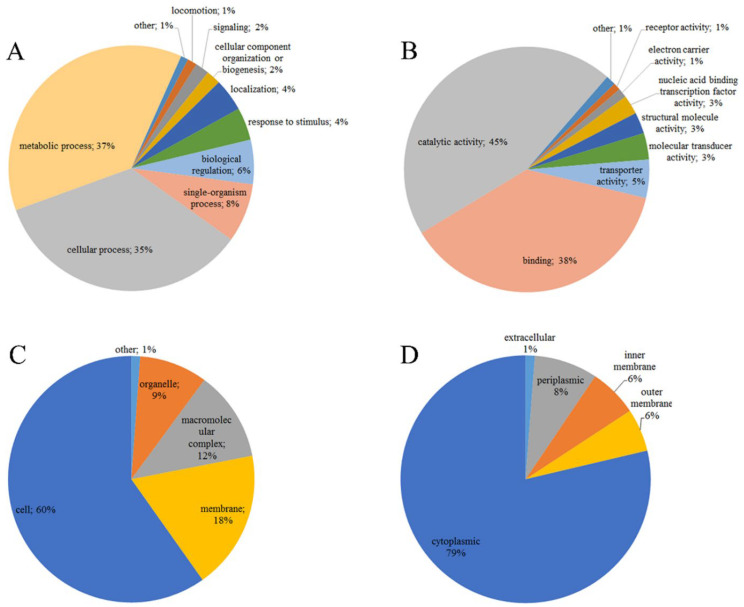
Gene ontology functional classification of the identified acetylation proteins based on (**A**) biological processes, showing the biological activities and physiological pathways these modified proteins participate in; (**B**) molecular function, describing the biochemical catalytic or binding functions exerted by acetylated proteins at the molecular level; (**C**) cell component, representing the subcellular structures, complexes and organelles where acetylated proteins reside inside bacterial cells; and (**D**) subcellular location, illustrating the quantitative proportion of acetylated proteins distributed in different subcellular compartments of *V. vulnificus* MO6-24/O.

To gain deeper insights into the functional significance of lysine acetylome in *V. vulnificus*, GO annotation was conducted to categorize all acetylated proteins (Figure 3, Supplementary Table S5).

In terms of both biological processes and molecular functions, enzymes involved in metabolism emerged as the predominant targets of acetylation, representing 37% and 45% of the identified proteins, respectively (Figure 3A,B). This predominance of metabolism-associated enzymes is in agreement with findings reported in earlier acetylome investigations [44,45,46]. The second most abundant group in biological processes was related to cellular processes, comprising 35% of the identified proteins (Figure 3A). From a molecular function perspective, another major group consisted of binding proteins, comprising 38% of the dataset (Figure 3B). Overall, the GO result indicates that lysine acetylation in *V. vulnificus* modulates a diverse array of physiological activities. According to the results of GO annotations, the acetylated proteins were predominantly localized to the cell (60%), with smaller proportions to the membrane (18%) and macromolecular complexes (12%) (Figure 3C). Because protein localization is closely linked to protein function [46,47], we conducted an examination of the subcellular distribution of the acetylated proteins in detail. The majority of the acetylated proteins were in the cytoplasm (79%), whereas smaller numbers were distributed in the periplasm (69 proteins), inner membrane (53 proteins), outer membrane (47 proteins), and extracellular environment (10 proteins) (Figure 3D). The distribution pattern aligns with previous acetylome profiles reported for *E. coli*, *V. parahaemolyticus*, and *H. mediterranei* [44,45,46] and is consistent with the GO-based functional classifications.

### 3.4. Functional Enrichment of Lysine Acetylome of V. vulnificus

**Figure 4:**
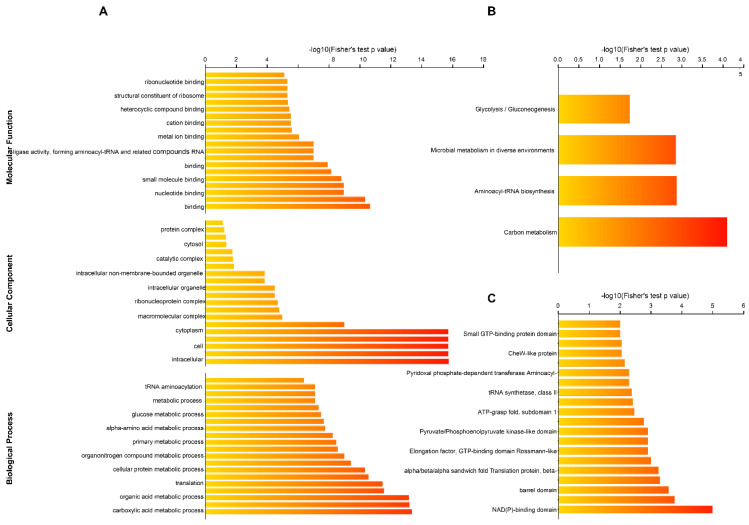
Enrichment analysis of the acetylated proteins in *V. vulnificus*. (**A**) A GO-based enrichment analysis of the acetylated proteins in terms of biological process, molecular function and cell component. (**B**) KEGG pathway-based enrichment analysis, displaying the top significantly enriched metabolic, virulence and physiological signaling pathways regulated by protein acetylation. (**C**) Protein domain-based enrichment analysis, illustrating the structural domains that are preferentially modified by lysine acetylation in *V. vulnificus* MO6-24/O.

To determine the classes of proteins that are preferentially modified by lysine acetylation, enrichment analyses were performed across three GO categories (Figure 4A). Consistent with previous findings [47,48], our results suggested that the acetylated proteins of *V. vulnificus* were predominantly enriched in metabolism and biosynthesis. Molecular function-based enrichment analysis revealed that proteins involved in binding interactions and ligase-related catalytic functions were more frequently subject to acetylation. GO cellular component analysis indicated that a large fraction of acetylated proteins was predominantly enriched in intracellular compartments, the cell, and the cytoplasm. Furthermore, KEGG enrichment analysis demonstrated that acetylated proteins associated with carbon metabolism, metabolism in diverse microbial environments, glycolysis and gluconeogenesis, as well as aminoacyl–tRNA biosynthesis pathways, were highly enriched (Figure 4B). These findings were further supported by domain enrichment analysis (Figure 4C). Protein domains associated with NAD(P)/ATP/small GTP-binding domains, the Pyruvate/Phosphoenolpyruvate kinase-like domain, Translation protein, aminoacyl–tRNA synthetase, Elongation factor, etc. were significantly enriched. Analysis of the enriched protein domains suggested that acetylation preferentially occurs on proteins harboring domains associated with energy metabolism and biosynthesis.

### 3.5. Acetylated Lysine Motifs in V. vulnificus

**Figure 5:**
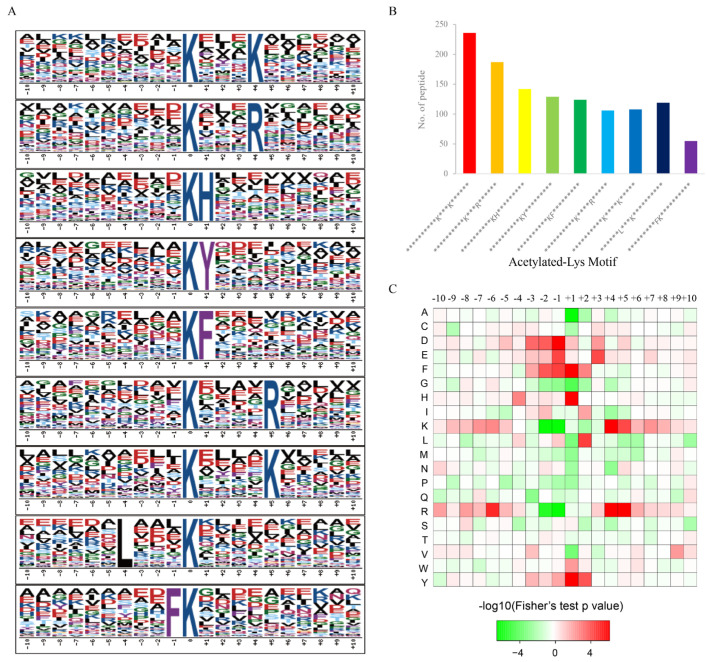
Properties of acetylated peptides. (**A**) Acetylation motifs and conservation of acetylation sites, generated via motif-x software using all full-length protein sequences from the database as background parameters. (**B**) Number of identified peptides containing acetylated lysine in each motif. Quantitative statistics displaying the total count of acetyl-lysine-containing peptides assigned to each distinct conserved acetylation motif. (**C**) Heat map of the amino acid compositions around the lysine acetylation sites, showing the frequency of different types of amino acids surrounding acetylated lysine.

Earlier investigations in prokaryotic systems have demonstrated that specific amino acid residues are preferentially enriched at positions flanking acetylated lysine [47,48,49,50]. To obtain a deeper understanding of the characteristics of lysine acetylation in *V. vulnificus*, sequence motif analysis was performed on all detected acetylated lysine sites with the Motif-X computational tool [51]. The amino acid sequences encompassing 10 residues upstream and downstream of each acetylation site were examined, resulting in the identification of nine clearly conserved motifs among 1206 acetylated peptides. These motifs included KacK, KacR, KacH, KacY, KacF, KacR, KacK, LxxKac, and FKac (Figure 5A), each displaying distinct frequencies of occurrence (Figure 5B). In these motifs, Kac represents an acetylated lysine residue, whereas the asterisk denotes any amino acid. Among the identified motifs, K^ac^xxxK was notably well-conserved, with roughly 19.57% of the acetylated peptides carrying this sequence feature. An analysis of all motifs indicated that residues flanking the acetylated lysine could be broadly divided into two major types: positively charged residues, including lysine (K), arginine (R), and histidine (H), and aromatic residues, such as phenylalanine (F) and tyrosine (Y).

Based on both the positional distribution and physicochemical properties of these surrounding residues, the motifs were further grouped into 2 categories: motifs favoring long-side-chain basic amino acids (K or R) at positions +4 or +5, and motifs enriched in bulky residues (Y, H, or F) in the immediate vicinity (−1 to +1) of the acetylated lysine (Figure 5C). Moreover, analysis of the amino acid composition heat map revealed that K and R were least frequently represented at positions −2 to +2 (Figure 5C). Interestingly, several acetylated lysine motifs have also been reported in prokaryotic species, including K^ac^xxxxK, K^ac^xxx K, K^ac^xxxxR, K^ac^xxxR, K^ac^H, K^ac^Y, in *V. parahaemolyticus*, *M. tuberculosis* and *S. roseosporus* [47,49,50], K^ac^xxxxR, K^ac^Y, K^ac^H, K^ac^F and FK^ac^ [48]. K^ac^xxxxK, K^ac^xxxK, K^ac^xxxxR, K^ac^xxxR, K^ac^Y and FK^ac^ were identified in *S. eriocheiris* [52], and K^ac^H was reported in *H. mediterranei* [53]. The presence of these motifs across diverse prokaryotic taxa suggests that lysine acetyltransferases are evolutionarily conserved and widely distributed among prokaryotes. This conclusion is consistent with earlier BLAST-based studies, which demonstrated the broad occurrence of lysine acetyltransferases in bacterial genomes [54,55]. However, a small subset of prokaryotic species has only yielded one detectable conserved acetylation motif (LxxxKac) in published acetylome datasets to date. This restricted motif diversity is probably due to the limited scale of lysine acetylation datasets currently available for prokaryotes, which may be inadequate for reliably identifying additional conserved sequence patterns. Collectively, these interspecies differences in motif repertoires suggest that the sequence context of acetylated lysines in *V. vulnificus* may have organism-specific features. However, motif analysis alone does not provide direct evidence for acetyltransferase substrate specificity.

### 3.6. Protein Interaction Networks of Acetylated Proteins in V. vulnificus

**Figure 6:**
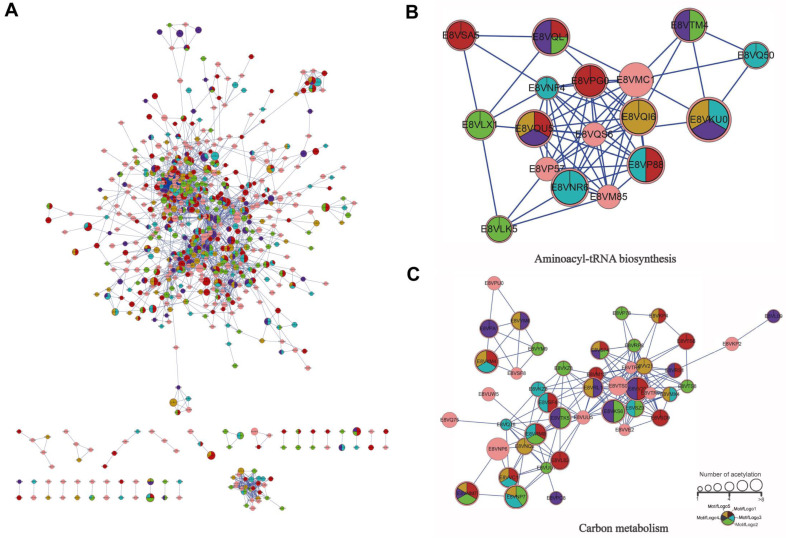
Protein interaction networks of acetylated proteins in *V. vulnificus*. (**A**) Interaction networks of the acetylated proteins in *V. vulnificus*. Nodes represent individual acetylated proteins, and edges indicate predicted functional and physical interactions between protein pairs, revealing the overall interactive relationships and core hub proteins of the acetylation regulatory network. (**B**) Subnetwork in aminoacyl–tRNA biosynthesis, exhibiting the interactive correlation of acetylation-modified proteins participating in amino acid activation and protein translation processes. (**C**) The enriched subnetwork of acetylated proteins associated with the carbon metabolism pathway, showing the key interactive acetylated proteins that dominate bacterial carbon utilization, energy metabolism and central metabolic regulation.

To gain deeper insight into the cellular pathways influenced by protein acetylation in *V. vulnificus*, a protein–protein interaction network was constructed for the identified acetylated proteins in the Cytoscape platform [41]. A global interaction network was constructed using 841 acetylated proteins as nodes (Figure 6A), including 2923 direct physical interactions from the STRING database that exhibited confidence scores exceeding 0.70. This network offers a comprehensive perspective on the roles of acetylated proteins in *V. vulnificus*, illustrating how they contribute to diverse biological processes. On the basis of this interaction network, several acetylation-associated protein complexes were identified. Furthermore, two densely connected modules of acetylated proteins were extracted by applying the MCODE in Cytoscape. The first cluster we detected (17 acetylated proteins) consisted of proteins related to Aminoacyl–tRNA biosynthesis (Figure 6B). The 17 acetylated proteins belong to ligases that participate in the biosynthesis of proteins. A dense protein–protein interaction network has been reported for acetylated proteins involved in Aminoacyl–tRNA biosynthesis, as observed in both *E. coli* and *V. parahemolyticus* [44,49]. The other subnetwork (44 acetylated proteins) was related to the carbon metabolism process (Figure 6C). This process mainly constitutes proteins from glycolysis/gluconeogenesis, pentose phosphate pathway (PPP) and citrate cycle. These findings indicated that lysine acetylation extensively targets proteins associated with aminoacyl–tRNA biosynthesis and carbon metabolism. A similar subnetwork enriched for primary metabolic pathways was also constructed for *S. eriocheiris* and *H. mediterranei* [52,53]. The network of protein interactions indicates that lysine acetylation plays a notably active role across diverse biological pathways in *V. vulnificus*. We next performed functional enrichment-based clustering for the acetylation motifs (Supplementary Figure S2). The GO enrichment analysis revealed that the five acetylation motifs are widely involved in biological processes and molecular functions in *V. vulnificus*, and motifs 1, 2 and 4 are mainly distributed in the cellular component.

### 3.7. Metabolic Pathways of Acetylated Proteins in V. vulnificus

**Figure 7:**
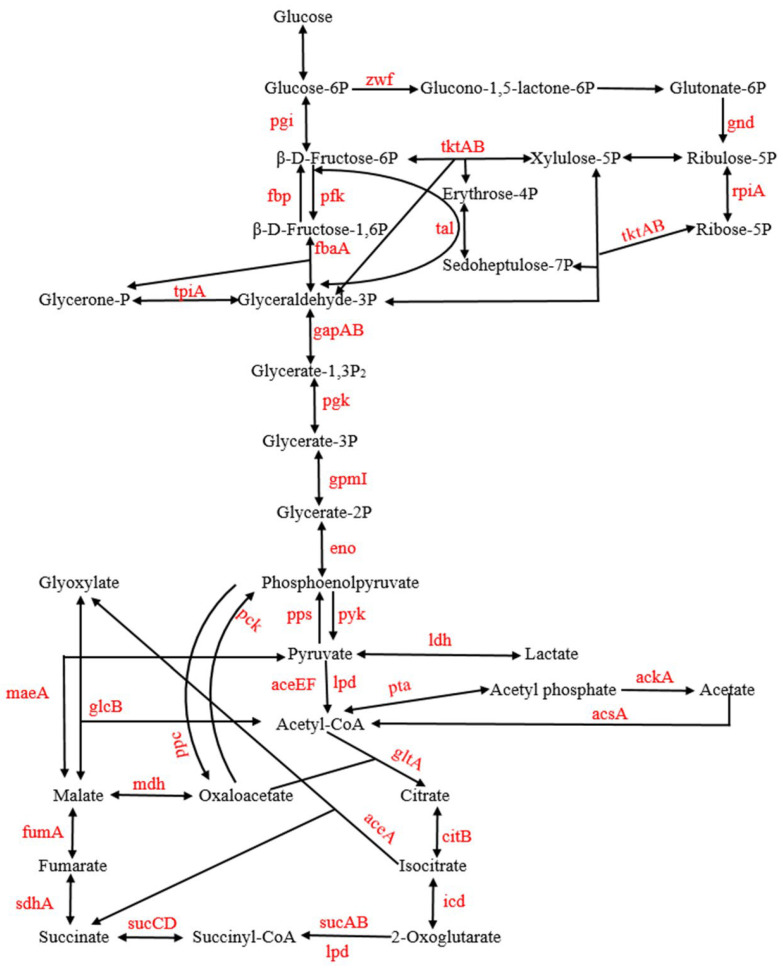
Schematic illustration of lysine-acetylation events identified in central metabolism of *V. vulnificus*. Lysine-acetylated enzymes are marked in red. This schematic systematically summarizes the distribution and modification sites of acetylated metabolic enzymes across central carbon metabolism, intuitively demonstrating the widespread regulatory roles of protein lysine acetylation in governing the fundamental metabolic network, energy homeostasis, and physiological metabolism of *V. vulnificus*.

Lysine acetylation serves as a key regulator of carbon flux within central metabolic pathways [43]. Consequently, this modification is widely regarded as a critical mechanism for the regulation of cellular metabolism [56]. However, current data on acetylated metabolic enzymes are very limited [44,52]. Consequently, this limitation hinders a thorough investigation into how lysine acetylation regulates metabolism in *V. vulnificus*. Acetylation modifications were observed in nearly all enzymes participating in central metabolic pathways (Figure 7). A significant portion of the acetylated proteins identified in glycolysis/gluconeogenesis, the TCA cycle, and the PPP are also known to be acetylated in other organisms such as *B. subtilis*, *E. coli*, *S. erythraea*, *Fragaria vesca*, *S. cerevisiae*, *S. eriocheiris*, *H. capsulatum*, and human cells. This cross-species conservation indicates that acetylation possibly serves as a preserved regulatory mechanism, potentially modulating the activity or stability of these key metabolic enzymes [57]. Notably, twelve acetylated enzymes participating in glycolysis/gluconeogenesis were identified in this study. For instance, glyceraldehyde-3-phosphate dehydrogenases GapA and GapB, which independently govern glycolysis and gluconeogenesis [58] were identified with acetylation modifications at four distinct sites each. Similarly, phosphoenolpyruvate carboxykinase, a critical rate-limiting enzyme in gluconeogenesis, was acetylated at seven different sites. Phosphoenolpyruvate carboxylase catalyzes the reaction between phosphoenolpyruvate and carbon dioxide to produce oxaloacetic acid, which catalyzes an irreversible anaplerotic reaction, and was acetylated at five sites. The pyruvate dehydrogenase complex, which serves as a critical metabolic bridge connecting glycolysis to the TCA cycle, was found to be extensively acetylated. Modifications were detected at thirty-nine distinct sites across all three of its constituent subunits. Furthermore, lysine acetylation was frequently observed among enzymes constituting the TCA cycle. For instance, acetylation sites were identified on the α-ketoglutarate dehydrogenase complex. The succinyl–CoA synthetase, which is the only reaction in the citric acid cycle to generate the high-energy phosphates, was acetylated at 6 sites. In the pentose phosphate pathway, many enzymes were acetylated. Glucose-6-phosphate dehydrogenase, together with trans-aldolase and transketolase, which link glycolysis to the PPP, were all acetylated. Within core metabolic pathways, four enzymes responsible for substrate-level phosphorylation, namely 6-phosphofructokinase, phosphoglycerate kinase, pyruvate kinase, and acetate kinase, were identified as targets of acetylation. In addition, acetylation was observed across all 12 dehydrogenases, indicating that this modification is widespread among key metabolic enzymes (Figure 7). Acetylation was also detected in four enzymes: glucose-6-phosphate isomerase, ribose-5-phosphate isomerase A, triosephosphate isomerase, and phosphoglucomutase. Enolase, a key glycolytic enzyme that also functions as a bacterial infection-related protein, exhibited acetylation at ten distinct sites [18,19,20]. Acetylation was detected on key enzymes responsible for acetate production and utilization, namely phosphate acetyltransferase, acetyl–coenzyme A synthetase, and acetate kinase. Taken together, the enzymes shown in Figure 7 suggest that lysine acetylation may play a regulatory role in metabolic enzymes responsible for the production of the cofactors NADH and ATP, thereby enabling cellular metabolism to adapt to changes in metabolic state [3].

## 4. Discussion

In this study, we identified 2035 acetylation sites on 841 proteins in *V. vulnificus* MO6-24/O. These modified proteins account for 18.5% of the total proteome. This suggests that lysine acetylation is a common modification in *V. vulnificus* and may play a role in its physiological regulation.

Functional annotation using the VFDB database identified 132 proteins associated with diverse virulence functions in *V. vulnificus* (Supplementary Table S6). Five core virulence factors (VvhA, OmpU, HlyU, RtxA, and IlpA) were screened with acetylation sites. These acetylated proteins modulate *V. vulnificus* virulence and may be associated with reversible modification, which regulates protein activity and stability, HlyU-dependent virulence gene transcription, acetyl–CoA-mediated metabolism–virulence coupling, host–pathogen interactions, and environmental adaptation [25]. In addition, the QS-related protein LuxS and the core QS regulator LuxO were also detected in this strain; acetylated LuxO participates in Vibrio QS regulation [59], while LuxS mediates bacterial pathogenesis via modulating QS signaling [60]. Further verification of *V. vulnificus* MO6-24/O revealed key virulence molecules: regulators HlyU and RpoS controlling RtxA toxin expression [29,61], hemolysin/cytolysin VvhA, and adhesion-related OmpU and IlpA that facilitate host infection [62,63].

Our data show that enzymes involved in central carbon metabolism are primary targets for acetylation, which is consistent with findings in other bacteria [19,20,24]. The identification of 39 acetylation sites on the PDH complex and multiple sites on Acs and CobB indicates that acetylation is involved in the management of carbon flux. This modification may allow *V. vulnificus* to adjust its metabolic activity when moving between estuarine environments and human hosts [2,36]. Furthermore, the clustering of acetylated proteins in aminoacyl–tRNA biosynthesis suggests that acetylation could influence protein synthesis, a pattern also observed in *E. coli* and *V. parahaemolyticus* [19,20].

Notably, several proteins related to virulence and host interaction, including OmpU, IlpA, and enolase, were found to be acetylated. Enolase, which has 10 modification sites in this study, is known to participate in bacterial infection and surface-related functions [39,40,41]. The acetylation of these proteins may affect their activity or localization during the infection process. Additionally, motif analysis showed a preference for basic residues (K/R) at the +4/+5 positions and aromatic residues (Y/H/F) at the −1/+1 positions. Similar motifs have been reported in other prokaryotes [24,28,30], reflecting a degree of conservation in the substrate specificity of bacterial acetyltransferases.

In summary, this study provides a systematic map of the *V. vulnificus* acetylome. The results indicate that lysine acetylation is widely distributed across proteins involved in metabolism, translation, and pathogenesis. These findings offer a basis for future research to examine the specific functions of these modifications in *V. vulnificus*.

This study has several limitations. First, growth-stage mixed samples were used for acetylome profiling without phase-specific analysis, yielding only static acetylation data and restricting the interpretation of dynamic acetylation regulation in *V. vulnificus*. Second, acetylation validation of Acs, CobB, OmpU and IlpA was performed via plasmid overexpression. While this approach confirmed protein acetylation and supported our proteomic results, overexpression disturbs native protein levels and acetylation homeostasis, which may not fully recapitulate in vivo modification states. Direct endogenous detection was unfeasible due to the extremely low basal abundance of these proteins in wild-type *V. vulnificus*. Third, despite the clinical origin of strain MO6-24/O, all acetylome analyses were performed using in vitro marine-cultured bacteria. Given the host cue-dependent nature of bacterial acetylation, the identified virulence protein acetylation profiles may differ from those occurring during actual human infection.

## 5. Conclusions

In this work, we systematically profiled the lysine acetylome of *V. vulnificus* MO6-24/O through high-sensitivity proteomics and Western blot validation. We identified 2035 acetylation sites on 841 proteins, representing 18.5% of the proteome. The acetylated proteins were widely localized among different cellular compartments and were predominantly associated with pathways involved in protein synthesis and carbon metabolic processes. Motif analysis showed that acetyl–lysine residues were commonly flanked by basic residues, lysine (K) or arginine (R), at the +4/+5 positions, or by bulky residues, tyrosine (Y), histidine (H), or phenylalanine (F), at the −1/+1 positions. Protein-interaction network analysis further indicated that lysine acetylation modulates a wide range of cellular processes including aminoacyl–tRNA biosynthesis, carbon metabolism (glycolysis/gluconeogenesis, the TCA cycle, and the PPP), energy production, and protein synthesis. Overall, this work advances understanding of acetylation-mediated regulation and provides a foundation for dissecting its roles in metabolism and virulence.

## Data Availability

The acetylome data of *V. vulnificus* strain MO6-24/O used in this study have been deposited in a public database and assigned the accession number OMIX017556. The data are available online via the corresponding database through the HTTP protocol.

## Supplementary Materials

The following supporting information can be downloaded at https://www.mdpi.com/article/10.3390/pathogens15070718/s1, Figure S1: Mass error distribution of identified peptides; Figure S2: GO enrichment clustering of acetylation motifs; Table S1: The plasmids and strains used in this study; Table S2: The primers used in this study; Table S3: Statistical table of acetyltransferases and deacetylases encoded in the *V. vulnificus* MO6-24/O genome; Table S4: The 841 acetylated proteins in the *V. vulnificus* MO6-24/O; Table S5: Categorization of all acetylated proteins by GO annotation; Table S6: Statistical analysis and annotation of *V. vulnificus* virulence factor genes in the VFDB database.

## Abbreviations

The following abbreviations are used in this manuscript:

PTM	Post-translational modification
Acs	Acetyl-CoA synthetase
CobB	NAD^+^-dependent protein deacetylase
OmpU	Outer membrane protein U
IlpA	Immunogenic lipoprotein A
GO	Gene Ontology
KEGG	Kyoto Encyclopedia of Genes and Genomes
