# Supplementary figures and images for: Acetylome Analysis in Vibrio vulnificus MO6-24/O Reveals Extensive Lysine Acetylation in Carbon Metabolism and Protein Synthesis Pathways: A Pilot Study
Source: Pathogens. 2026 Jul 7;15(7):718. doi: 10.3390/pathogens15070718 (PMC13415618; doi:10.3390/pathogens15070718)

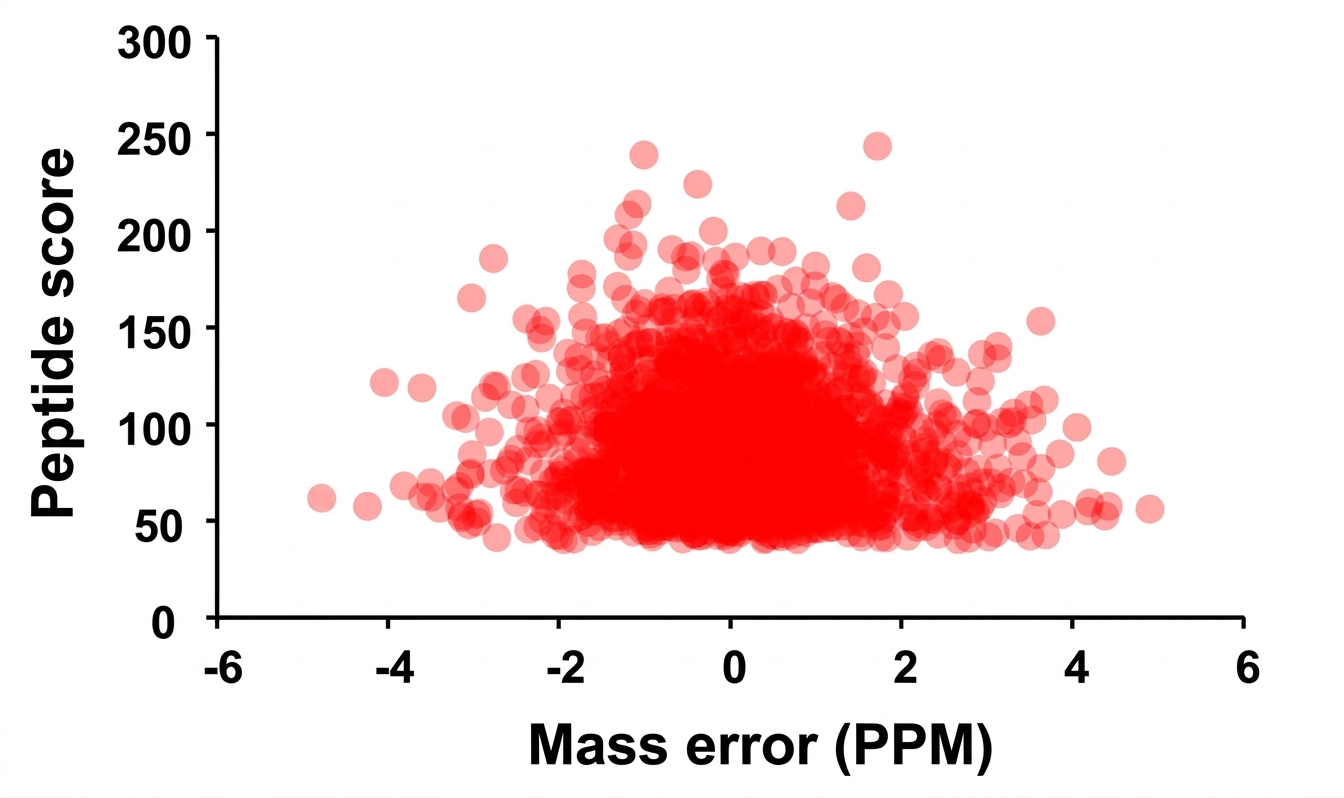

Supplement: Supplementary file 1 [file pathogens-15-00718-s001.zip › Figure S1.tif]

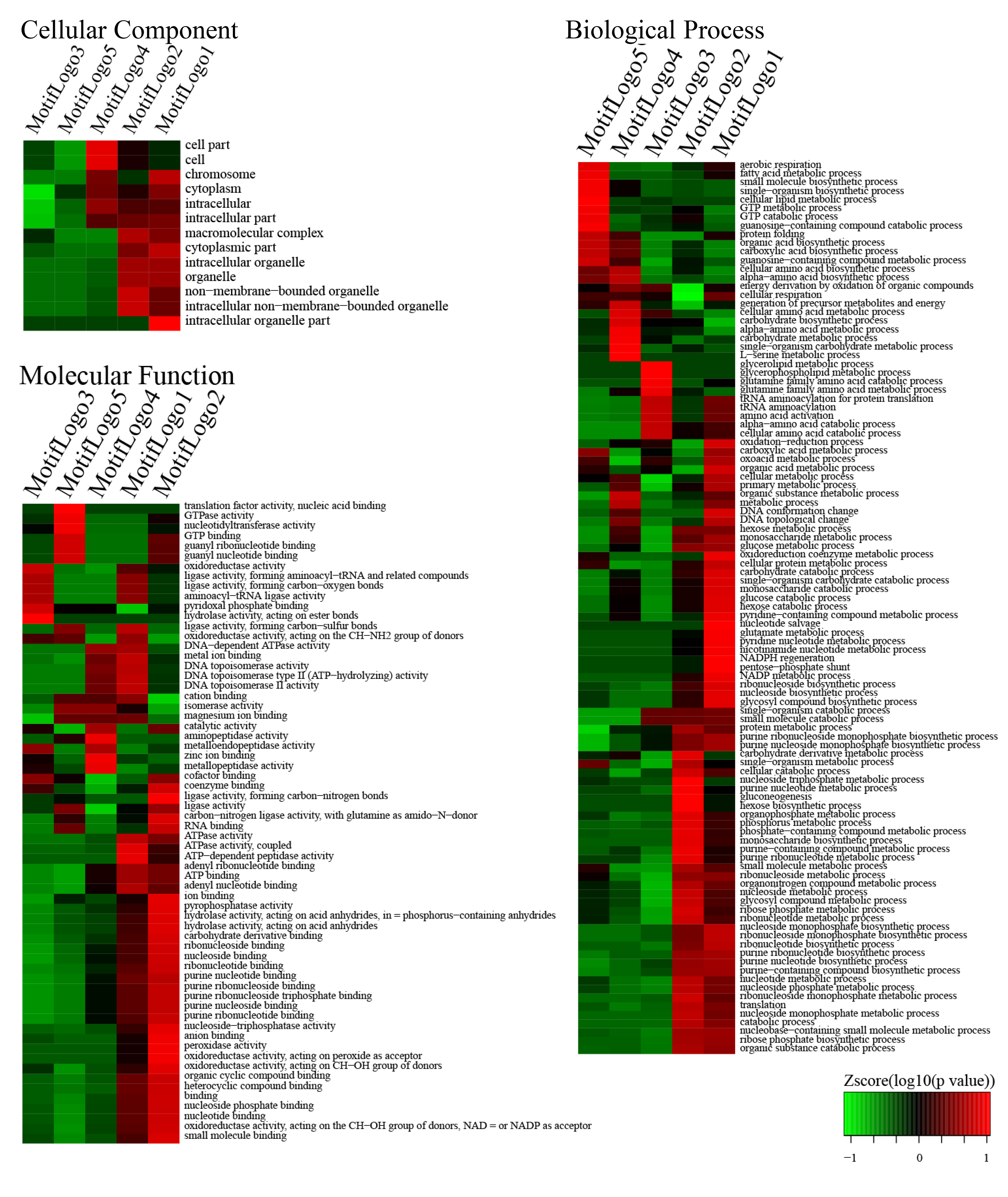

Supplement: Supplementary file 1 [file pathogens-15-00718-s001.zip › Figure S2.tif]

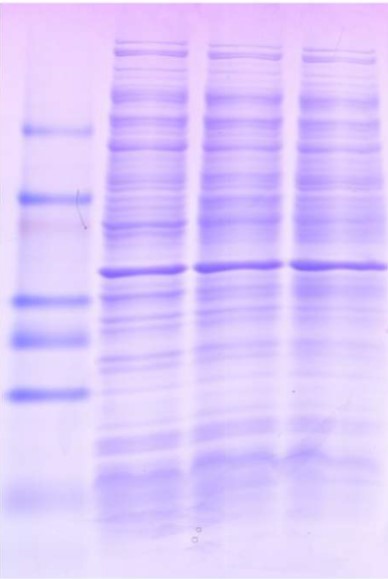

Supplement: Supplementary file 1 [file pathogens-15-00718-s001.zip › Original Western Blot Images/Figure1A(Unlabled).tiff]

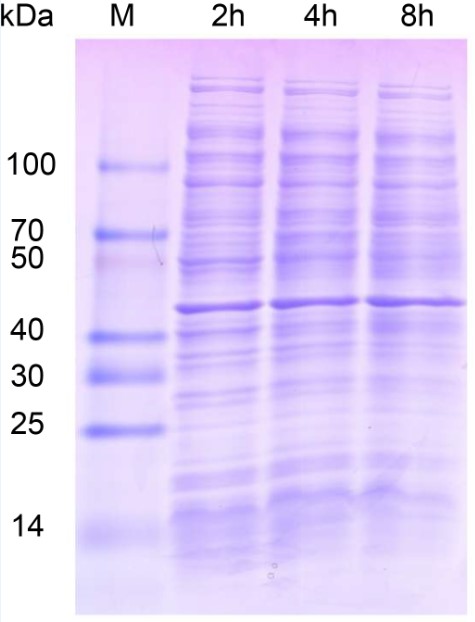

Supplement: Supplementary file 1 [file pathogens-15-00718-s001.zip › Original Western Blot Images/Figure1A.tiff]

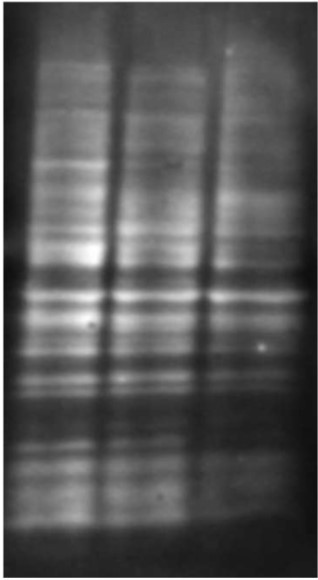

Supplement: Supplementary file 1 [file pathogens-15-00718-s001.zip › Original Western Blot Images/Figure1B.tiff]

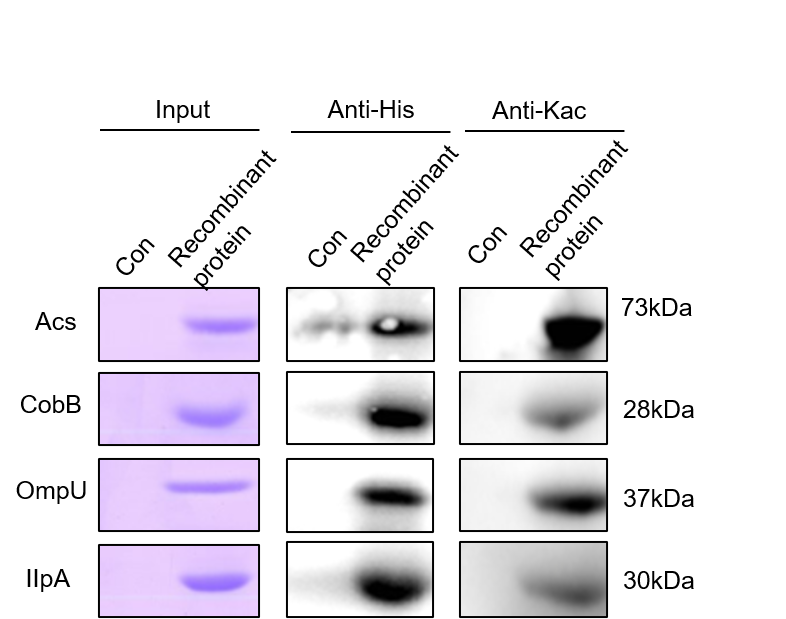

Supplement: Supplementary file 1 [file pathogens-15-00718-s001.zip › Original Western Blot Images/Figure2 (in text).tif]

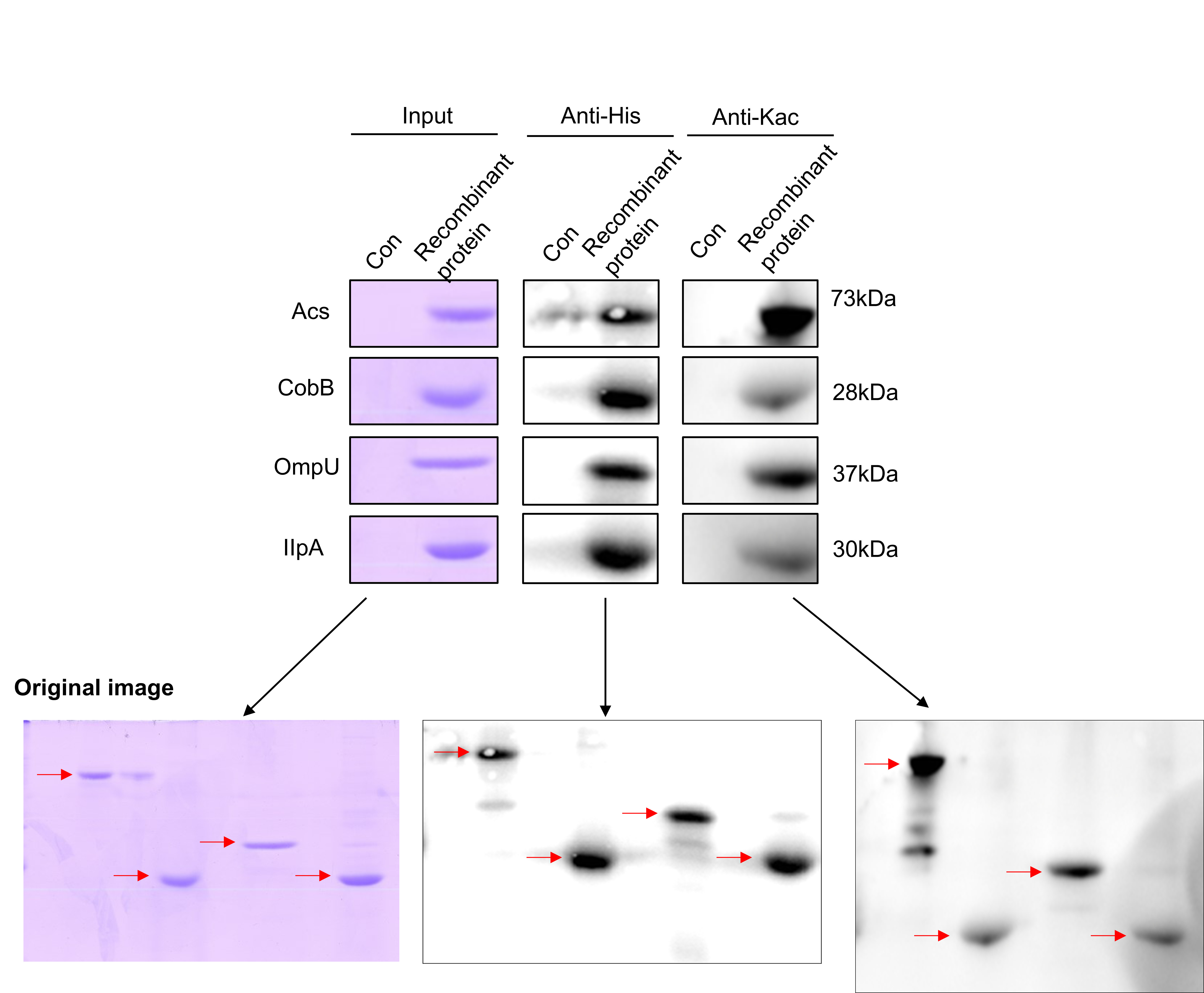

Supplement: Supplementary file 1 [file pathogens-15-00718-s001.zip › Original Western Blot Images/Figure2+original image.tif]

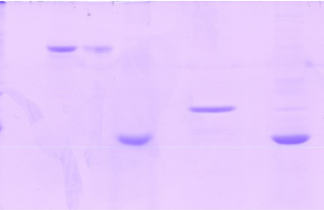

Supplement: Supplementary file 1 [file pathogens-15-00718-s001.zip › Original Western Blot Images/Figure2-1(Unlabled).tif]

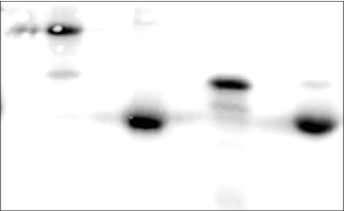

Supplement: Supplementary file 1 [file pathogens-15-00718-s001.zip › Original Western Blot Images/Figure2-2(Unlabled).tif]

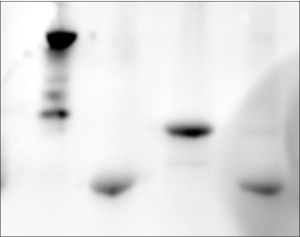

Supplement: Supplementary file 1 [file pathogens-15-00718-s001.zip › Original Western Blot Images/Figure2-3(Unlabled).tif]
